# Hypomineralized Second Primary Molars as Predictor of Molar Incisor Hypomineralization

**DOI:** 10.1038/srep31929

**Published:** 2016-08-25

**Authors:** A. Negre-Barber, J. M. Montiel-Company, M. Boronat-Catalá, M. Catalá-Pizarro, J. M. Almerich-Silla

**Affiliations:** 1 Departament d’Estomatologia, Facultad de Medicina i Odontologia, Universitat de València, Spain.

## Abstract

Molar incisor hypomineralization (MIH) is a developmental defect of dental enamel that shares features with hypomineralized second primary molars (HSPM). Prior to permanent tooth eruption, second primary molars could have predictive value for permanent molar and incisor hypomineralization. To assess this possible relationship, a cross-sectional study was conducted in a sample of 414 children aged 8 and 9 years from the INMA cohort in Valencia (Spain). A calibrated examiner (linear-weighted Kappa 0.83) performed the intraoral examinations at the University of Valencia between November 2013 and 2014, applying the diagnostic criteria for MIH and HSPM adopted by the European Academy of Paediatric Dentistry. 100 children (24.2%) presented MIH and 60 (14.5%) presented HSPM. Co-occurrence of the two defects was observed in 11.1% of the children examined. The positive predictive value was 76.7% (63.9–86.6) and the negative predictive value 84.7% (80.6–88.3). The positive likelihood ratio (S/1-E) was 10.3 (5.9–17.9) and the negative likelihood ratio (1-S/E) 0.57 (0.47–0.68). The odds ratio was 18.2 (9.39–35.48). It was concluded that while the presence of HSPM can be considered a predictor of MIH, indicating the need for monitoring and control, the absence of this defect in primary dentition does not rule out the appearance of MIH.

The European Academy of Paediatric Dentistry (EAPD)^1^ has defined molar incisor hypomineralization (MIH) as being a defect in the mineralization of one to four permanent first molars, sometimes associated with similarly affected permanent incisors, that is presented as demarcated opacities of variable extent and severity. They have clear borders, can be white, yellow or brown in color and can develop into post-eruptive enamel breakdown (PEB) and extensive atypical caries, and may even require extraction.

MIH and HSPM are probably caused by a disturbance during the initial calcification and/or maturation of the enamel of the affected teeth^2^,^3^. Because of the temporal association between the mineralization of the crowns of the permanent first molars and that of the second primary molars, these could also be affected by hypomineralization if some factor were to operate during this developmental stage, particularly during the prenatal and perinatal period^4^.

Hypomineralization has been observed in second primary molars and even in the cusps of permanent second molars and canines^1^,^5^,^6^. Hypomineralized Second Primary Molars (HSPM)^7^ is the term currently used for the condition previously known as Deciduous Molar Hypomineralisation (DMH)^4^. Both describe the same idiopathic hypomineralization that affects between one and four second primary molars.

The characteristic features of HSPM are the same as for MIH: opaque stains that vary in tone between white, yellow and brown, post-eruptive enamel breakdown, atypical restorations and/or extensive caries with opacities at the margins, sensitivity, tenderness and difficult treatment^4^. Opacities are considered the mild form of MIH and HSPM, and atypical extractions the most severe form^8^.

The considerable variability in the criteria for designing and conducting MIH and HSPM prevalence surveys leads to highly disparate results. A literature review by Elfrink *et al*.^9^ found prevalence ranges of 2.9% to 44% for MIH and 0% to 21.8% for HSPM. Consequently, a standard protocol for prevalence and etiology studies of these defects has been proposed in order to allow comparison of the results and achieve higher standards of evidence^10^.

The current evidence on the etiology of MIH and HSPM is very weak. There is no clear cause-and-effect link to pre- or peri-natal diseases such as illness during pregnancy, medicine use during pregnancy, premature birth or underweight at birth. However, illnesses during the first three years of life, particularly fever, asthma or pneumonia, could have an effect on MIH^11^. Some authors have suggested that the cause could be a genetic variation^12^.

To date, it has not proved possible to establish a causal relationship with any particular pathological agent and the appearance of the defect cannot be prevented, so identifying factors that may be predictors of MIH is relevant to tackling this disorder. Since second primary molars erupt four years before permanent first molars, the aim of this study was to assess the predictive value of HSPM for the appearance of MIH, as well as their distribution in the child population of Valencia (Spain).

## Materials and Methods

### Ethical Approval

Written informed consent was obtained from all participants’ parents or legal guardians and the study procedures were clearly explained to the parents/guardians and participants before their inclusion. The study was conducted according to Declaration of Helsinki principles for medical research involving human subjects. The clinical study was approved by the University of Valencia’s human research ethics committee, under procedure number H1372162226937. The methods were carried out in accordance with the approved guidelines.

### Study design

This cross-sectional study was part of a wider research project named INMA (the Spanish initials for infancy, childhood and the environment), which investigates the effects of environmental factors on the development and health of a cohort of children in the Valencia region of Spain whose mothers were recruited by consecutive sampling in early pregnancy and have been followed-up since their inclusion in 2003–2004^13^,^14^.

### Sample size calculation

A minimum sample size of 405 subjects was calculated as sufficient to estimate, with a 95% confidence level and a precision of +/− 4%, an MIH population percentage considered to be around 20%. A replacement rate of 5% was anticipated.

### Study population

The final sample comprised 414 children aged 8 and 9 years (born between 2004 and 2005) in the Valencia region of Spain enrolled in the INMA project.

### Calibration of the examiner

The first step was to standardize the diagnostic criteria for MIH/HSPM and prepare a data collection form. These were reviewed and discussed by the examining dentist and an experienced professional who was considered the gold standard.

The first calibration session was conducted with 46 clinical photographs which were used to assess all the degrees of hypomineralization as well as other disorders involved in a differential diagnosis, such as fluorosis, hypoplasia and amelogenesis imperfecta. The diagnostic agreement was 100%. A second calibration session was then carried out with 54 children who attended the pediatric dentistry unit in the stomatology department of the University of Valencia, distinguishing between sound teeth, mild MIH/HSPM and severe MIH/HSPM. The agreement measured by linear-weighted Kappa was 0.83, which is considered a good result on the Landis and Koch scale. A pilot study was conducted to clarify or correct any errors that had arisen.

### Examination

The examinations were carried out at the pediatric dentistry unit in the stomatology department of the University of Valencia. The equipment comprised two dental chairs with lighting, cotton swabs for removing excess plaque or saliva, flat mouth mirrors and sterilized standard no. 4/6 double-ended exploration probes.

The data were collected on an examination record specifically prepared for this study, i which had a section for personal details and a dental chart for recording the data on MIH and HSPM.

The diagnostic criteria for MIH were those drawn up by the EAPD^1^ in 2003 and adapted for HSPM in 2008^4^, in both cases as revised in 2015^9^,^10^. They are as follows: white-yellow-brown demarcated opacities, post-eruptive enamel breakdown (PEB) associated with opacities, atypical extensive caries with surrounding opacities or in low-risk surfaces, atypical restorations of a size and location unrelated to the caries pattern, crowns if MIH is found in other teeth, and extractions due to MIH. Stains greater than 1 mm were counted as hypomineralized. The occlusal/incisal, labial and lingual/palatine surfaces of the permanent incisors, permanent first molars and second primary molars were assessed. The teeth were examined when wet.

MIH was diagnosed when a permanent first molar was affected by hypomineralization and HSPM was diagnosed when at least one second primary molar showed any of the characteristics described for MIH. The incisors were only diagnosed as MIH if a molar was affected. The degree of severity was decided by the most severe disorder in the child’s mouth. White, creamy/yellow or dark brown opacities were counted as mild MIH/HSPM. Post-eruptive enamel breakdown, extensive caries with surrounding opacities and atypical restorations, crowns or extractions due to MIH were counted as severe MIH/HSPM.

### Statistical analysis

The completed examination records were entered into an Access® data base (Access 2003; Microsoft Corporation, Redmont, WA, USA) and transferred to an Excel® spreadsheet (Excel 2003; Microsoft Corporation, Redmont, WA, USA) for treatment by the SPSS Statistics 22.0® program (IBM SPSS, Chicago, IL, USA).

Descriptive statistics were calculated, with means and confidence intervals for the quantitative variables and percentages and confidence intervals for the qualitative variables. Student’s t-test or ANOVA were used to determine significant differences between means and a chi-square test to detect differences between percentages. To evaluate HSPM as a diagnostic test for MIH, a sensitivity and specificity analysis was conducted and the predictive values, likelihood ratio and odds ratio were also calculated. The level of significance was set at p < 0.05.

## Results

A total of 414 children were examined (212 boys and 202 girls). The mean age of the sample was 9.16 years (95% CI 9.13–9.18).

The data on MIH and HSPM prevalence, degrees of severity and number of teeth affected are shown in Table 1. A total of 24.2% (100 children) presented MIH, 72% to a mild degree and 28% severe, so one in four children was affected by MIH and one in four of the children with MIH was severely affected. HSPM was observed in 14.5% (60 children), to a mild degree in 91.7% and severe in 8.3% of the cases. No statistically significant differences between boys and girls was observed for either MIH, found in 54% of the boys and 46% of the girls (chi-square test p = 0.521), or HSPM, found in 53.3% and 46.7% respectively (chi-square test p = 0.722). Nor was any association encountered between gender and MIH severity (chi-square test p = 0.343).

A combination of affected molars and incisors was more frequent than molars alone: of the 100 children with hypomineralization of permanent teeth, 40 (40%) had molar hypomineralization (MH) alone and 60 (60%) presented molar incisor hypomineralization (MIH).

The mean number of teeth affected by hypomineralization was 2.77 permanent first molars, 1.29 permanent incisors and 1.96 second primary molars. In general, maxillary teeth were affected more than mandibular teeth and the right side more than the left, but the differences were not statistically significant (Table 1). The teeth of each type most often affected were the upper right permanent first molar, upper center-right incisor and upper right second primary molar. By severity, the worst-affected teeth were the upper right permanent first molar and lower left second primary molar. No severe cases of hypomineralization were found in the permanent incisors.

Among the children with MIH, the mean number of surfaces with hypomineralization was 5.93 (95% CI 5.24–6.61). The mean number of hypomineralized second primary molar surfaces was 0.13 (95% CI 0.05–0.20) in the children without MIH (n = 314) and 1.62 (95% CI 1.18–2.05) in those with MIH (n = 100).

Analyzing a cross table for HSPM as a diagnostic test for MIH (Table 2) gave 46% (35.9–56.2) sensitivity and 95.5% (92.6–97.5) specificity. Its positive predictive value was 76.7% (63.9–86.6) and its negative predictive value 84.7% (80.6–88.3). The positive likelihood ratio (SE/1-SP) was 10.3 (5.9–17.9) and the negative likelihood ratio (1-SE/SP) was 0.57 (0.47–0.68). The odds ratio was 18.2 (9.39–35.48).

## Discussion

HSPM prevalence varies in different countries, ranging from 2.9% to 21.8%^2^,^4^,^7^,^15^,^16^,^17^,^18^,^19^. The HSPM prevalence in the present study was 14.5% (60 of the 414 children examined). The HSPM prevalence was lower than that of MIH, which agrees with the findings of other authors^2^,^4^,^7^,^16^,^19^.

MIH prevalence around the world ranges from 2.9% to 44%^6^. In Spain, MIH prevalence studies have shown levels of between 12.4% and 21.8%^20^,^21^,^22^. The present study found a slightly higher prevalence level: 24.2%.

The definition of MIH specifies that a permanent first molar must always be affected, but not necessarily an incisor. However, Balmer *et al*.^23^ observed hypomineralization affecting only incisors (IH) in 11% of cases and Schamalfuss *et al*.^6^ observed hypomineralized canines in 22.8% of children with MIH. The interval between the onset of mineralization in permanent first molars and in incisors is very short, so they could be expected to be similarly affected^24^. In the present study, the permanent first molars and incisors were both affected in 60% of the cases of MIH, while in 40% only the molars showed hypomineralization (MH). This tendency is also found in other authors^21^,^22^,^25^,^26^,^27^.

As regards the severity of MIH and HSPM, MIH is most often encountered in a mild form^6^,^16^,^21^,^22^,^27^,^28^,^29^,^30^,^31^. Approximately one in four cases of MIH is severe. This agrees with the results of the present study, where 72% of the MIH cases were mild and 28% severe. MIH most frequently appears in more than one molar/child^3^,^6^,^20^,^21^,^22^,^26^,^27^,^28^,^32^ and the average number of teeth affected ranges between 2 and 5.7. In the present study the mean number of teeth/child affected by MIH was 4, of which 2.77 were first molars (2.27 in a mild form and 0.5 severely) and 1.29 were permanent incisors (all with mild hypomineralization).

For HSPM the picture is similar, with the mild form predominating in the present study (92%), as in others^4^,^7^,^16^. The mean number of second primary molars affected was 1.96/child, of which 1.86 were mild and 0.1 severe. Similar results were obtained by Elfrink *et al*.^4^, with a mean of 1.9, and Mittal and Sharma^16^. with a mean of 2.47 teeth.

The teeth most often affected were the upper right permanent first molar, upper center-right incisor and upper right second primary molar. In terms of severity, the teeth most affected by severe hypomineralization were the upper right permanent first molar and lower right second primary molar. No significant association was found between gender and MIH/HSPM.

Co-occurrence of MIH and HSPM was observed by Temilola *et al*.^17^ in 34.8% of cases and by Mittal and Sharma^16^ in 32.7%. Elfrink *et al*.^2^ observaron que un 26,5% de los niños con HSPM también tenian MIH. Ghanim *et al*.^7^ found that 39.6% of the children with HSPM suffered MIH. Da Costa-Silva *et al*.^19^ observed that 5.2% of the children with HSPM had MIH, but encountered no significant differences. In the present study, 11.11% of the sample (total n = 414) had both MIH and HSPM. Of the children with HSPM (n = 60), 76% had MIH.

Elfrink *et al*.^2^ obtained a 4.4 odds ratio for HSPM in the children with MIH, differentiating between mild (odds ratio of 5.3) and severe (odds ratio of 4.0) HSPM. This would indicate that those with HSPM are at greater risk of suffering MIH than HSPM-healthy children. A recent study by Mittal and Sharma^16^ obtained an odds ratio of 7.82 and that of the present study is still higher, at 18.2 (95% CI 9.39–35.38).

In order to ascertain the true extent of MIH and HSPM with any precision, comparable representative studies need to be conducted using the same methods. The present study followed the criteria established by EAPD^1^, which agree with those proposed at the 12^th^ EAPD Congress held in Sopot (Poland) in 2014, published by Elfrink *et al*.^9^ and Ghanim *et al*.^10^. The main researcher’s calibration Kappa score ensured correct diagnosis of both MIH and HSPM.

A total of 414 children were examined for this study. The examinations were performed with the child in a dental chair to ensure reproducibility of the conditions in other studies. The recommended age for MIH studies is eight to nine years^3^ and that for HSPM is five years^4^. The mean age of the present study sample was 9.16 years, but the error in estimating HSPM owing to early exfoliation, extensive caries or crowns is considered small, since 97% of the children still possessed all their second primary molars and only a small percentage of the primary molars examined (2.5%) could not be assessed.

In order to discover whether the presence of HSPM can be used as a predictor of MIH, the data from the 414 children in the cohort were analyzed as a diagnostic study. Low sensitivity (0.46) and high specificity (0.95) were obtained. The explanation for the low sensitivity is that only 46% of the MIH patients presented HSPM. The high specificity is because 95.4% of those without MIH did not present HSPM either. Neither sensitivity nor specificity are useful in clinical practice, however, since the eruption of the primary second molar occurs approximately 4 years before that of the permanent incisors and first molars. For this reason, it is more important to discover positive and negative predictive values.

On examining the predictive values it was found that when a child was diagnosed with HSPM, the probability of his or her having MIH was 76.7% (positive predictive value) and when no HSPM was present the probability of not suffering MIH was 84.7% (negative predictive value). Since positive and negative predictive values are influenced by the prevalence of the process they aim to predict, it is considered more appropriate to present the likelihood ratios, which do not suffer from this drawback. The positive likelihood ratio of 10.3 indicates high predictive power for MIH, as it shows that the probability of suffering MIH is 10.3 times greater in those affected by HSPM than in those who are not affected by it. However, the negative likelihood ratio of 0.57 indicates a lower capacity to predict the absence of MIH in the children without HSPM. The probability of not suffering MIH is only 1.75 times greater in those unaffected by HSPM than in those affected by this condition.

The clinical applicability of diagnosing the presence of HSPM is based on the high probability that children suffering from this condition will present MIH, and consequently should be monitored and included in high risk caries control groups. MIH is of considerable concern to clinicians because the permanent first molars are a key developmental element and can be affected by eruption problems. Incisors are more easily diagnosed but molars are overlooked by patients and/or their parents and are detected at a late stage. As second primary molars erupt about four years before permanent first molars, early diagnosis and monitoring will allow more preventive measures to be taken, applying them at an early stage to teeth with MIH.

To conclude, the presence of HSPM can be considered a predictive factor for MIH, although the absence of this defect in the primary dentition does not rule out the future appearance of MIH. The predictive factor provided by HSPM indicates the need to monitor these patients and check them at more frequent intervals.

## Additional Information

**How to cite this article**: Negre-Barber, A. *et al*. Hypomineralized Second Primary Molars as Predictor of Molar Incisor Hypomineralization. *Sci. Rep.* **6**, 31929; doi: 10.1038/srep31929 (2016).

## Figures and Tables

**Table 1:** Distribution of Hypomineralized Second Primary Molars and Molar Incisor Hypomineralization in the sample (n = 414).

	Molar Incisor Hypomineralization (MIH) n = 100		Hypomineralized Second Primary Molars (HSPM) n = 60
**Prevalence** % of total sample	24.2% (20.2–28.5)		14.5% (11.4–18.2)
**Degree of severity** % within MIH or HSPM	Mild = 72% (62.5–79.9)		Mild = 91.7% (81.9–96.3)
	Severe = 28% (20.1–37.4)		Severe = 8.3% (3.6–18.1)
**Teeth affected** mean	**First molars** 2.77 (2.54–2.99) Mild = 2.27 Severe = 0.5	**Incisors** 1.29 (1.01–1.56) Mild = 1.29	**Second primary molars** 1.96 (1.72–2.24) Mild = 1.86 Severe = 0.1
**No. of teeth affected** 1 tooth 2 teeth 3 teeth >or = 4 teeth	**First molars** 17% (10.8–25.5) 22% (15.0–31.1) 26% (18.4–35.3) 35% (26.4–44.7)	**Incisors** 25% (17.5–34.3) 12% (6.9–19.8) 13% (7.7–20.9) 10% (5.5–17.4)	**Second primary molars** 35% (24.2–47.6) 41.7% (30.1–54.3) 15% (8.1–26.1) 8.3% (3.6–18.1)
**Prevalence per tooth** % of total sample	**First molars** 1.6 = 18.6% (15.1–22.6) 2.6 = 17.8% (14.4–21.8) 3.6 = 14.5% (11.4–18.2) 4.6 = 15.9% (12.7–19.7)	**Incisors** 1.2 = 2.8% (1.6–4.9) **1.1** = 8.7% (6.3–11.8) **2.1** = 6.5% (4.5–9.3) **2.2** = 2.7% (1.5–4.6) **3.2** = 3.1% (1.8–5.3) **3.1** = 1.9% (0.9–3.7) **4.1** = 3.4% (2.0–5.6) **4.2** = 2.4% (1.3–4.4)	**Second primary molars** 5.5 = 8.9% (6.5–12.1) **6.5** = 7.5% (5.3–10.4) **7.5** = 3.9% (2.3–6.1) **8.5** = 6.8% (4.7–9.6)

**Table 2:** Cross table of Hypomineralized Second Primary Molars (HSPM) as a predictive test for Molar Incisor Hypomineralization (MIH) (n = 414).

		Molar Incisor Hypomineralization (MIH)		
					No	Yes
**Hypomineralized Second Primary Molars (HSPM)**	**No**	TN = 300 NPV = 84.7% SP = 95.5%	FN = 54	354
	**Yes**	FP = 14	TP = 46 PPV = 76.7% SE = 46.0%	60
		314	100	n = 414

TN true negative, FN false negative, NPV negative predictive value, SP specificity, FP false positive, TP true positive, PPV positive predictive value, SE sensitivity.

## References

[b1] Weerheijm K. L. . Judgement criteria for molar incisor hypomineralisation (MIH) in epidemiologic studies: a summary of the European meeting on MIH held in Athens, 2003. Eur J Paediatr Dent. 4, 110–113 (2003). 14529329

[b2] Elfrink M. E. . Deciduous molar hypomineralization and molar incisor hypomineralization. J Dent Res. 91, 551–555 (2012). 22370445 10.1177/0022034512440450

[b3] Weerheijm K. L. Molar incisor hypomineralisation (MIH). Eur J Paediatr Dent. 4, 114–120 (2003). 14529330

[b4] Elfrink M. E., Schuller A. A., Weerheijm K. L. & Veerkamp J. S. Hypomineralized second primary molars: prevalence data in Dutch 5-year-olds. Caries Res. 42, 282–285 (2008). 18523388 10.1159/000135674

[b5] Dietrich G., Sperling S. & Hetzer G. Molar incisor hypomineralisation in a group of children and adolescents living in Dresden (Germany). Eur J Paediatr Dent. 4, 133–137 (2003). 14529334

[b6] Schmalfuss A., Stenhagen K. R., Tveit A. B., Crossner C. G. & Espelid I. Canines are Affected in 16-Year-Olds with Molar-Incisor Hypomineralisation (MIH): An Epidemiological Study Based on the Tromso Study: “Fit Futures”. Eur J Paediatr Dent. 17, 107–113 (2016). 26683199 10.1007/s40368-015-0216-6

[b7] Ghanim A., Manton D., Marino R., Morgan M. & Bailey D. Prevalence of demarcated hypomineralisation defects in second primary molars in Iraqi children. Int J Paediatr Dent. 23, 48–55 (2013). 22276809 10.1111/j.1365-263X.2012.01223.x

[b8] Weerheijm K. L., Jalevik B. & Alaluusua S. Molar-incisor hypomineralisation. Caries Res. 35, 390–391 (2001). 11641576 10.1159/000047479

[b9] Elfrink M. E., Ghanim A., Manton D. J. & Weerheijm K. L. Standardised studies on Molar Incisor Hypomineralisation (MIH) and Hypomineralised Second Primary Molars (HSPM): a need. Eur Arch Paediatr Dent. 16, 247–255 (2015). 25894247 10.1007/s40368-015-0179-7

[b10] Ghanim A., Elfrink M., Weerheijm K., Marino R. & Manton D. A practical method for use in epidemiological studies on enamel hypomineralisation. Eur Arch Paediatr Dent. 16, 235–246 (2015). 25916282 10.1007/s40368-015-0178-8 PMC4469791

[b11] Silvia M. J., Scurrah K. J., Craig J. M., Manton D. J. & Kilpatrick N. Etiology of Molar Incisor Hypomineralization - A Systematic Review. Community Dent Oral Epidemiol. 44, 342–353 (2016) 27121068 10.1111/cdoe.12229

[b12] Vieira A. R. & KUP E. On the Etiology of Molar-Incisor Hypomineralization. Caries Res. 50, 166–169 (2016). 27111773 10.1159/000445128

[b13] Guxens M. . Cohort Profile: the INMA–INfancia y Medio Ambiente–(Environment and Childhood) Project. Int J Epidemiol. 41, 930–940 (2012). 21471022 10.1093/ije/dyr054

[b14] Ribas-Fito N. . Child health and the environment: the INMA Spanish Study. Paediatr Perinat Epidemiol. 20, 403–410 (2006). 16911019 10.1111/j.1365-3016.2006.00745.x

[b15] Elfrink M. E., Veerkamp J. S., Aartman I. H., Moll H. A. & Ten Cate J. M. Validity of scoring caries and primary molar hypomineralization (DMH) on intraoral photographs. Eur Arch Paediatr Dent. 10, 5–10 (2009). 19863892 10.1007/BF03262693

[b16] Mittal N. & Sharma B. B. Hypomineralised second primary molars: prevalence, defect characteristics and possible association with Molar Incisor Hypomineralisation in Indian children. Eur Arch Paediatr Dent. 16, 441–447 (2015). 26092507 10.1007/s40368-015-0190-z

[b17] Temilola O. D., Folayan M. O. & Oyedele T. The prevalence and pattern of deciduous molar hypomineralization and molar-incisor hypomineralization in children from a suburban population in Nigeria. BMC Oral Health. 30, 15:73 (2015). 10.1186/s12903-015-0059-x PMC4486434 26121979

[b18] Ng J. J., Eu O. C., Nair R. & Hong C. H. Prevalence of molar incisor hypomineralization (MIH) in Singaporean children. Int J Paediatr Dent. 25, 73–78 (2015). 24602187 10.1111/ipd.12100

[b19] Costa-Silva C. M., Paula J. S. d., Ambrosano G. M. B. & Mialhe F. L. Influence of deciduous molar hypomineralization on the development of molar-incisor hypomineralizarion. Braz J Oral Sci. 12, 335–338 (2013).

[b20] Comes-Martínez Á., Puente-Ruiz C. & Rodríguez-Salvanés F. Prevalencia de Hipomineralización en primeros molares permanentes (MIH) en población infantil del Área 2 de Madrid. RCOE. 12, 129–134 (2007).

[b21] Martinez-Gomez T. P., Guinot-Jimeno F., Bellet-Dalmau L. J. & Giner-Tarrida L. Prevalence of molar-incisor hypomineralisation observed using transillumination in a group of children from Barcelona (Spain). Int J Paediatr Dent. 22, 100–109 (2012). 21883558 10.1111/j.1365-263X.2011.01172.x

[b22] Garcia-Margarit M., Catala-Pizarro M., Montiel-Company J. M. & Almerich-Silla J. M. Epidemiologic study of molar-incisor hypomineralization in 8-year-old Spanish children. Int J Paediatr Dent. 24, 14–22 (2014). 23317396 10.1111/ipd.12020

[b23] Balmer R., Toumba K. J., Munyombwe T., Godson, & Duggal & The Prevalence of Incisor Hypomineralisation and its Relationship with the Prevalence of Molar Incisor Hypomineralisation. Eur Arch Paediatr Dent. 16, 265–269 (2015). 25894246 10.1007/s40368-014-0171-7

[b24] Mejare I., Bergman E. & Grindefjord M. Hypomineralized molars and incisors of unknown origin: treatment outcome at age 18 years. Int J Paediatr Dent. 15, 20–28 (2005). 15663441 10.1111/j.1365-263X.2005.00599.x

[b25] Muratbegovic A., Zukanovic A. & Markovic N. Molar-incisor-hypomineralisation impact on developmental defects of enamel prevalence in a low fluoridated area. Eur Arch Paediatr Dent. 9, 228–231 (2008). 19054477 10.1007/BF03262640

[b26] Jasulaityte L., Weerheijm K. L. & Veerkamp J. S. Prevalence of molar-incisor-hypomineralisation among children participating in the Dutch National Epidemiological Survey (2003). Eur Arch Paediatr Dent. 9, 218–223 (2008). 19054475 10.1007/BF03262638

[b27] Lygidakis N. A., Dimou G. & Briseniou E. Molar-incisor-hypomineralisation (MIH). Retrospective clinical study in Greek children. I. Prevalence and defect characteristics. Eur Arch Paediatr Dent. 9, 200–206 (2008). 19054473 10.1007/BF03262636

[b28] Jalevik B., Klingberg G., Barregard L. & Noren J. G. The prevalence of demarcated opacities in permanent first molars in a group of Swedish children. Acta Odontol Scand. 59, 255–260 (2001). 11680642 10.1080/000163501750541093

[b29] Leppaniemi A., Lukinmaa P. L. & Alaluusua S. Nonfluoride hypomineralizations in the permanent first molars and their impact on the treatment need. Caries Res. 35, 36–40 (2001). 11125194 10.1159/000047428

[b30] Calderara P. C. . The prevalence of Molar Incisor Hypomineralisation (MIH) in a group of Italian school children. Eur J Paediatr Dent. 6, 79–83 (2005). 16004536

[b31] Costa-Silva C. M. . Molar incisor hypomineralization: prevalence, severity and clinical consequences in Brazilian children. Int J Paediatr Dent. 20, 426–434 (2010). 20738434 10.1111/j.1365-263X.2010.01097.x

[b32] Muratbegovic A., Markovic N. & Ganibegovic-Selimovic M. Molar incisor hypomineralisation in Bosnia and Herzegovina: aetiology and clinical consequences in medium caries activity population. Eur Arch Paediatr Dent. 8, 189–194 (2007). 18076849 10.1007/BF03262595

